# A monthly global paleo-reanalysis of the atmosphere from 1600 to 2005 for studying past climatic variations

**DOI:** 10.1038/sdata.2017.76

**Published:** 2017-06-06

**Authors:** Jörg Franke, Stefan Brönnimann, Jonas Bhend, Yuri Brugnara

**Affiliations:** 1 Institute of Geography, University of Bern, 3012 Bern, Switzerland; 2 Oeschger Centre for Climate Change Research, University of Bern, 3012 Bern, Switzerland; 3 Federal Office of Meteorology and Climatology, Zürich Airport, 8058 Zürich, Switzerland

**Keywords:** Palaeoclimate, Atmospheric dynamics

## Abstract

Climatic variations at decadal scales such as phases of accelerated warming or weak monsoons have profound effects on society and economy. Studying these variations requires insights from the past. However, most current reconstructions provide either time series or fields of regional surface climate, which limit our understanding of the underlying dynamics. Here, we present the first monthly paleo-reanalysis covering the period 1600 to 2005. Over land, instrumental temperature and surface pressure observations, temperature indices derived from historical documents and climate sensitive tree-ring measurements were assimilated into an atmospheric general circulation model ensemble using a Kalman filtering technique. This data set combines the advantage of traditional reconstruction methods of being as close as possible to observations with the advantage of climate models of being physically consistent and having 3-dimensional information about the state of the atmosphere for various variables and at all points in time. In contrast to most statistical reconstructions, centennial variability stems from the climate model and its forcings, no stationarity assumptions are made and error estimates are provided.

## Background & Summary

Studying past decadal climate variations requires global, comprehensive data sets that are consistent with atmospheric dynamics. Ideally, such studies would be based on 3-dimensional, global representations of atmosphere and ocean in high resolution. Until recently, paleoclimatology has produced either single time series or spatially interpolated, paleodata-based reconstructions that allow to infer information mostly about past temperature and hydroclimatic conditions^1^. While paleoclimatology has learned tremendously from these data sets, they may not be suitable for studying decadal variations as they do not provide global coverage, are not comprehensive (lack important variables), and may not be physically consistent. Such physical consistency, however, can only come from climate model simulations.

In atmospheric sciences, reanalysis data sets that combine measurements with model simulations have become available in the 1990s and have revolutionized the field. Atmospheric reanalyses are the most widely used data sets in geosciences, with together tens of thousands of citations. While the Twentieth Century Reanalysis project^2^ and the European ERA-20C data set^3^ have shown that conventional reanalyses approaches can be extended back in time, there is a limit as to how far back the global weather can be captured. Here we present an approach that aims at a monthly time scale and exploits, at the same time, the information from early instrumental measurements, documentary data, paleodata such as tree rings, and climate model simulations.

**Figure 1:**
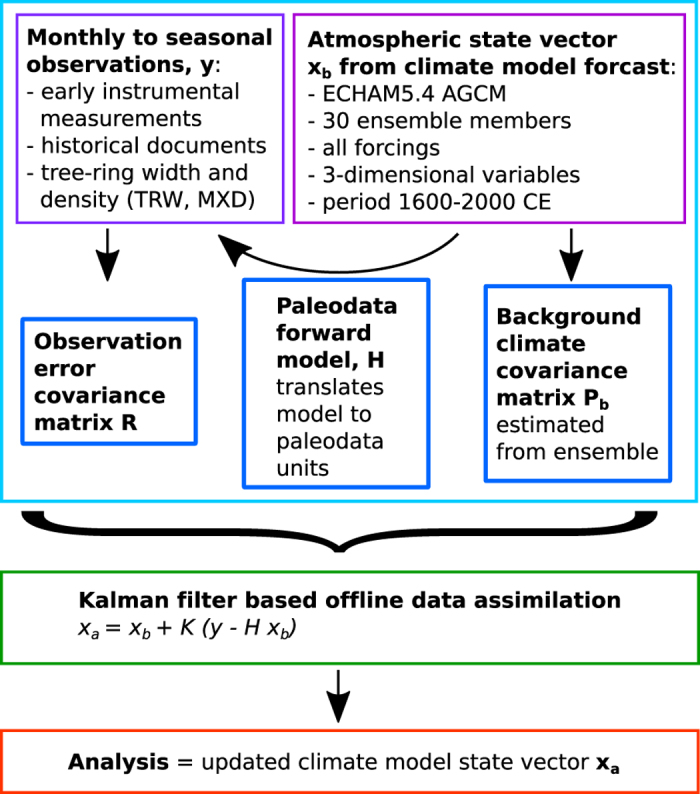
Schematic of the assimilation procedure.

The backward extension of conventional reanalyses was made possible by applying the Ensemble Square Root Filtering technique (EnSRF)^4^. Here we use a similar approach, but with a half-yearly assimilation step. We argue that with this assimilation frequency, initial conditions do not matter anymore, while the boundary conditions (sea-surface temperature, external forcings, etc.) provide some predictability. Therefore, the assimilation can be performed off-line on an ensemble of transient AGCM simulations (Fig. 1).

The main advantages responsible for the success of reanalysis over statistical reconstructions techniques are: i) the full spatial and temporal (4 dimensional) coverage; ii) the possibility to draw conclusions on model variables, which are not assimilated, e.g., only assimilating sea level pressure (SLP) can lead to meaningful temperature and precipitation patterns; iii) there are no assumptions about stationarity, a problem in most other reconstruction approaches because patterns may often change over time^5^. These are the reasons why researchers now start to investigate the potential of using even sparser and noisier paleo-climatic data to create a paleo-reanalysis^6–8^. Recently, first results from an annually resolved assimilation project were published that solely relies on proxy data, where single model years from existing simulations covering the last millennium are used as ensemble members without taking their actual model year into account^9^.

This study bridges the gap between the annually resolved analysis^9^ and analyses for the last 150 years^2,3^. It builds upon idealized model-world experiments^6^ and is the first monthly resolved paleo-reanalysis for the period 1600–2005. We assimilate early instrumental temperature and sea level pressure data, indices from historical documents as well as tree-ring width and density information into a 30-member ensemble with an atmospheric general circulation model. In the following sections, we will demonstrate the feasibility and potential of this new approach.

## Methods

Data assimilation provides a best estimate of the state of the atmosphere based on climate model physics and observations (we use ‘observations’ in the following to denote instrumental data, documentary data, and proxy data). There are errors (i.e., difference to the unknown truth) on both sides. The model boundary conditions do not fully constrain the result, but the model generates random weather consistent with the boundary conditions. Its error is expressed in the mean bias and in the spread of an ensemble of simulations. Observations also have errors, e.g., due to instrument changes, reporting errors, or non-climatic signals in proxies. Again, there are random and systematic errors. Assimilation techniques calculate the most likely state of the atmosphere given all errors in both, models and observations. Here, we explain the Ensemble Kalman Fitting assimilation method on which this study is based before we present the climate model and observations used.

### Ensemble kalman fitting (EKF) method

Mathematically, data assimilation can be expressed as a minimization of errors. The following cost function (1) is minimized:

(1)J(x)=(x−xb)T(Pb)−1(x−xb)+(y−H[x])TR−1(y−H[x])

where x is a vector of the true atmospheric state or a time average of the state^10^. *x*^*b*^ is the background or first guess, i.e., here represented by the raw model simulations. *P*^*b*^ is the model error covariance matrix, which we calculate from the ensemble of simulations. *R* describes the observation error covariance matrix. *y* are the observations and the operator *H* extracts the observations from the model space (see Experimental design and proxy forward model). In the following H denotes the Jacobian matrix of *H*(*x*).

Assuming normally distributed probabilities, this cost function can be minimized with a Kalman filter, where the best estimate of the true atmospheric state *x* is the analysis *x*^*a*^ given by equation (2):

(2)xa=xb+PbHT(HPbHTR)−1(y−Hxb)

We work with a sequential implementation of the Ensemble Kalman Filter^4^. This variation is computationally much less demanding and allows to update the ensemble members individually without explicitly updating the covariance matrices. To account for a bias occurring in the analysis covariance, we apply the ensemble square root filter^11^. Thereby, the assimilation procedure can be split into an update of the ensemble mean (denoted by $\bar{x}$) and an update of the anomalies about the ensemble mean (denoted by *x*′):

(3)x¯a=x¯b+K(y¯−Hx¯b)

(4)x′a=x′b+K˜(y′−Hx′b)=(I−K˜H)x′bwith:y′=0

with the Kalman gain matrices *K* and $\tilde{K}$^6,11^:

(5)K=PbHT(HPbHT+R)−1

(6)K˜=PbHT[(HPbHT+R)−1]T×(HPbHT+R+R)−1

In numerical weather prediction, the procedure is cycled, i.e., x_a_ becomes the initial conditions for the next simulation step. The model thus propagates the information further into the unobserved phase space. In a paleo-climatic setting, this is not the case. The initial state is not well defined by the sparse, noisy data available in the past and the model has no skill in predicting the subsequent month from initial conditions. Nevertheless, the model simulations have skill originating from the boundary conditions (see ‘data’ section). Since initial conditions do not matter, a cycle is not required. This allows us to first run the entire simulation and assimilate the data afterward (off-line assimilation) instead of assimilating data after each calculation time step and then continuing the simulation (on-line assimilation)^12^. We call this method, which is no data assimilation in the traditional way, Ensemble Kalman Fitting (EKF)^6^. The new reanalysis is called EKF400 because it covers the past 400 years.

### Covariance localization

Because of our finite and small ensemble size of 30 simulations we have to deal with spurious correlations in the background error covariance matrix *P^b^*. These lead to random, unphysical updates and an ‘over-correction’ of the analysis because very distant and in reality uncorrelated locations correlate by chance in the model world. To prevent this effect, we define a function to set a cut-off distance beyond which no update takes place following^6,13^:

(7)Pi,jb=1nens−1∑k=1nensxi,k′bxj,k′bexp(−|di−dj|22L2)

with *n*_ens_ different ensemble members. The absolute value of *d*_*i*_−*d*_*j*_ is the distance in km between grid box *i* and grid box *j*, and L is the cut-off distance. We estimate *L* in the model world by calculating decorrelation distances for each variable in the state vector. This distinction is necessary as temperature for example exhibits correlations over much larger regions than precipitation (Table 1).

The assimilation step is 6-months, i.e. we assimilate April-to-September and October-to-March seasons, which are the growing seasons influencing seasonal tree-ring measurements in the northern and southern hemisphere, respectively. However, we keep monthly data for all six months in the state vector. In this way, we can assimilate monthly instrumental and documentary observations as well as seasonal tree ring-based data and achieve a final analysis with monthly resolution. Combining six months into one state vector has been successfully done for assimilating total column ozone data into chemistry-climate model simulations using the same EKF method^14^. Our state vector thus has a length of *n*=304,164.

### Experimental design and proxy forward model

**Figure 2:**
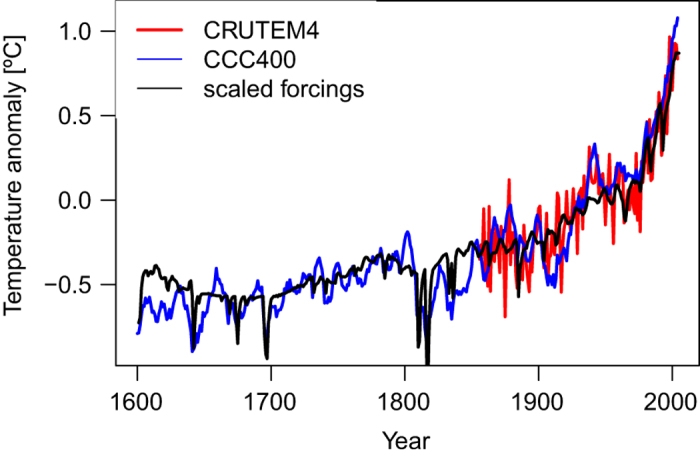
Global mean land temperature anomalies and scaled forcings. The agreement between instrumental measurements and simulations indicates that the model scales the external forcings to a realistic multi-decadal to centennial global mean temperature evolution: instrumental CRUTEM4 data set (red), the CCC400 simulations (blue) and the CCC400 forcings regressed to CRUTEM4 (black).

While R and P^b^ are suited to measure the random part of the error, systematic biases are more difficult to treat. The model may prefer a colder state over a given region, and as soon as observations become available, they pull the model towards them, leading to a step change. Biases may also occur in the boundary conditions. Likewise paleodata often suffer from uncertain multi-decadal to centennial variability^15^. We solve this problem by assimilating anomalies for 70-year periods around the current year, i.e., model anomalies are corrected by the assimilation of observation anomalies. As a result, variability on scales longer than 70-yrs in the analysis purely stems from the model, whereas seasonal to decadal variability is influenced by observational anomalies. To assess whether the model produces realistic multi-decadal to centennial variability, we compared CCC400 global mean land temperature with instrumental global mean temperatures from the CRUTEM4 ref.(16) and found good agreement (Fig. 2). Further, we produced a simple reconstruction of global mean land temperature from fitting 400-year time series of external forcings (CO_2_, solar irradiance, tropospheric aerosols and stratospheric optical depth) to CRUTEM4 in a multiple regression approach with an autoregressive term (Fig. 2). CCC400 is in very good agreement with the resulting curve. It is thus reasonable to assume that CCC400 realistically reproduces variability on time scales longer than 70 years.

Assimilation entails an operator *H* that mimics the observations in model space, called observation operator or forward operator. For instrumental data, this means extracting the corresponding grid cell and variable from the model state vector. In the case of documentary indices, which are given in standard deviations, additional standardising (in the 70-yr window) is required. Forward operators for the paleodata tree-ring width (TRW) and maximum latewood density (MXD) are more complex. Tree rings are often influenced by more than one factor, e.g., temperature and humidity^17^. Proxy forward models to simulate tree-ring width such as VS or VS-Lite^18^ are available and could in principle be used. We decided for this study to choose a simpler approach. We used a multiple regression approach that is informed by VS-Lite. First, all TRW series were modelled with VS-lite^19^. From this, the relevant variables were identified as temperature during 4 months JJAS (in the northern hemisphere) and precipitation during the 3 months AMJ. The TRW series were then calibrated against corresponding CRU TS3.10 data in a regression approach. For tree-ring density we use multiple regression only for monthly temperature during the growing season as density is hardly affected by precipitation^20,21, 22,23^. Regression coefficients are calculated for a common period of overlapping paleodata and instrumental data (1901–1960).

Although the reanalysis is monthly, for simplicity we mostly present semi-annual results averaged for boreal summer (AMJJAS) and winter (ONDJFM).

### Error/Uncertainty estimations

To exclude outliers from being assimilated, we first apply a variance screening by estimating local variance in the CCC400 simulations. Then, we check if the anomalies of the early instrumental data at the current time step is more than four standard deviations from the mean. If this is a case, we assume it to be an outlier and exclude it from the assimilation. This is justified because we do not expect extreme events such as hurricanes causing such strong deviations in monthly averages.

Estimating a specific error for each individual record that gets assimilated is practically impossible because information about data quality exists only for a few instrumental records that have been homogenized recently. For early instrumental data, all records that pass the screening get a common plausible error assigned (1K for temperature and 3 hPa for SLP). Error contributions come from various sources. One major contribution is usually assessed by data homogenization projects and includes errors such as shading of thermometers or uncertainties due to human reading of the instrument. Altogether, we estimate this part to be around 0.5 K based on expert knowledge. A second error source is stations not being representative for a grid box average. The latter error has been estimated by MeteoSwiss for one of their gridded data sets to be around 0.5 K in the non-alpine regions of Switzerland^24^. Hence, we believe an error of around 1 K to be a realistic first guess estimate.

Instrumental measurements can be assumed to be more precise than interpretations of historical documents but the documentary data is often only available as anomalies in units of standard deviations. Thus, we set the documentary data error to 0.5 standard deviations. Note that only documentary temperature information is assimilated.

For the tree-ring data we use the variance of the multiple regression residuals as an error estimate. i.e., if the tree-rings contain a strong climatic signal, the multiple-regression model will create a good fit to instrumental data and consequently the residuals will be small.

## Data Records

### Model simulations

In this study, we use the atmospheric general circulation model simulations^6^, which we call ‘CCC400’. This is a 30-member ensemble of ECHAM5.4 ref.(25) simulations at a triangular spectral truncation of T63 and 31 levels in the vertical covering the period 1600 to 2005.

The simulations are driven by reconstructed and observed monthly variations in aerosol optical depth caused by volcanic eruptions^26^ and variations in tropospheric aerosols^27^, total solar irradiance^28^, greenhouse gases^29^, land surface conditions^30^, sea ice cover and sea surface temperatures (SST). The reconstructed annual SSTs^31^ (SST_year mean_) are superimposed with a seasonal anomaly (SST_seas′_) and ENSO dependent anomalies (SST_ENSO′_), both based on HadISST1.1 ref.(32), with SST_ENSO’_ being estimated by least squares regression on the reconstructed annual El Niño 3.4 index^33^ with lag 0 and lag −1:

(8)SST=SSTyearmean+SSTseas′+SSTENSO′

For lack of a skillful sea-ice reconstruction, we use the sea-ice climatology from the HadISST1.1 data set before 1870. This is expected to result in a underestimation of high latitude climate variability in the simulations.

Unfortunately, land-surface changes have not been correctly included, which caused an overestimation of its effects on albedo, surface roughness, vegetation variables and field capacity. We assessed the bias with one corrected simulation that could be conducted additionally. Thirty-year mean temperatures over extra-tropical northern hemisphere (20–90°N) land areas are on average 0.28 K (−0.5 to +1.2 K locally) warmer at the beginning of the 17th century and are on average 0.31 K (−1 to +2.4 K locally) warmer in the late 20th century than the corrected simulation. Other variables such as pressure, wind and precipitation are also slightly affected. Since the model is debiased in the pre-processing scheme using a 70-yr window, effect of the land surface specification on the assimilation will be small (see ‘Experimental design’) for monthly to seasonal averages, which is the target of this study. Additionally, only around Antarctica, excessively high and unrealistic wind speeds are reached, which cause wave-like patterns poleward of 60° S. Therefore, we exclude any interpretation of the latitudes south of 60° S. To keep the assimilation computationally tractable we use every second grid box and only keep the variables seen in Table 2 in the state vector. Each variable has 4,608 grid boxes over land and ocean.

### Observations

**Figure 3:**
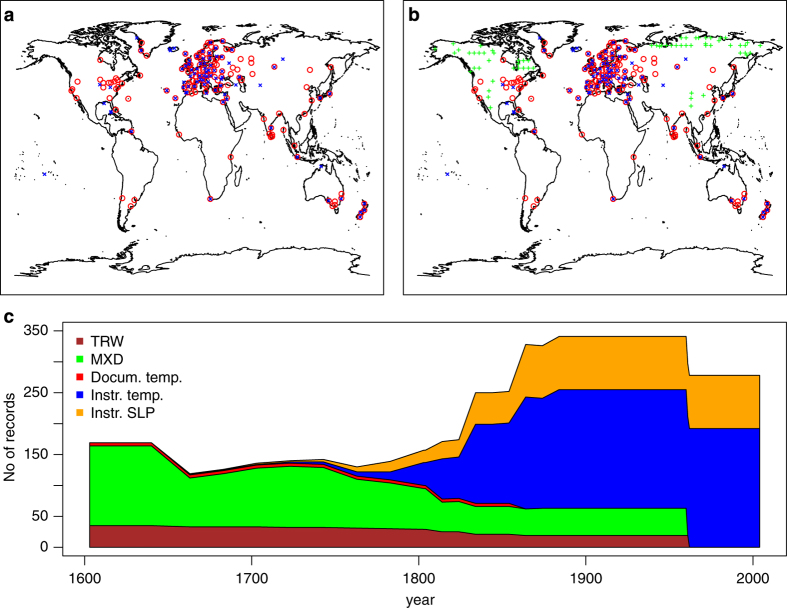
Spatial and temporal overview of assimilated data. Locations with instrumental temperature measurements are highlighted with red circles, sea level pressure stations with blue crosses and tree-ring width/density locations with green ‘plus’ symbol in October to March (**a**) and April to September (**b**) of the year 1880. The time dependent number of input data is shown in (**c**) separated by data type and variable.

We assimilate monthly instrumental measurements of temperature and sea level pressure, monthly temperature information from documentary sources and half-yearly tree-ring paleodata: tree-ring width (TRW) and maximum latewood density (MXD). We always use all information available (*m* records) except for instrumental data where we freeze the network at the year 1880 state, i.e., we exclude all series that start after 1880. Our main goal is to construct a paleo-reanalysis, for the 20th century there is already a higher resolved product available^2^. Thus, we use the 1880 data network during the 20th century mainly to evaluate the quality of our reanalysis in the past. The first instrumental series start in the year 1659, the last documentary series end in 1853 and all TRW and MXD records end in 1960 (Fig. 3).

Instrumental temperature series include the GHCN collection^34^ the collection of the Climate Research Unit at the University of East Anglia^35^, historical instrumental time series of the Greater Alpine Region (HISTALP)^36,37^, as well as temperature series from Central England^38^, Rio de Janeiro^39^, Funchal Madeira^40,41^, Nagasaki, Tokyo and Yokohama^42^, Illussat, Nuuk and Qaqortoq^43^ and 18th Century temperature measurements from Jamaica^44^. Instrumental SLP mainly comes from GHCN^45^ and HISTALP, too. Additionally, we incorporated a collection of European SLP measurements^46^, series from Salem Massachusetts^47^, GordonCastle^48^, Nagasaki and Tokyo^42^ and Nyzhny Tagil^49^. We use monthly resolved documentary temperature information for the Carpathian basin^50^, the Czech Republic^51^, Germany^52^, Poland^53^ and Switzerland^54^. We use 35 climate sensitive TRW records^8,19^. MXD records mainly stem from a previous collection^55^. Additionally, we use density records from the Austrian Alps^56^, Swiss Alps^57^, Carpathians^58^, Jeamtland^59^, Fjorfordalen^23^, Pyrenees^60^, Tatra^61^ and Tornetrask^21^.

In very few places in Europe and the United States of America we must deal with multiple time series that are located in the same model grid box. In these cases, we prefer instrumental series to documentary series to paleodata, i.e., if there is an instrumental measurement in a grid box, we discard the paleodata. If more series of the same type exist, we calculate a grid box average. All instrumental data are in the form of anomalies (see section ‘experimental design’). Therefore, biases, e.g., due to different elevations above sea level, can be neglected.

### Validation data sets

To assess the skill of our analysis we compare it with several observational data sets for the 20th century. We chose the gridded temperature, precipitation and SLP data sets CRU TS 3.10 ref.(62). To assess uncertainties in the gridded instrumental data sets, mostly due to measurement errors and interpolation, we consult the CRUTEM ensemble of instrumental fields^16^. Before 1900, we conduct a leave-one-out validation and compare our assimilation product to the spatial reconstruction^46,63,64^ for temperature, precipitation and SLP in Europe, respectively. Furthermore, we use several proxy based reconstructions of northern hemisphere land temperature^65,66^, calculated atmospheric circulation indices^67^ and reconstructed indices^68,69, 70,71, 72^ as well as the 20th century reanalysis^2^ version 2c (20CR) and European ReAnalysis ERA-40 ref.(73).

## Technical Validation

Estimating the skill of EKF400 is challenging because the network of assimilated data and hence the skill varies with each time step. Moreover, the goal would be to assimilate all data available, thus no completely independent data would be left for validation. For this reason, we present a set of skill measures: comparisons with 20th century instrumental observations, time series of large-scale average temperature from observations and paleodata. Finally, we present a case study for the 1815 Tambora eruption, which caused severe climatic conditions 200 years ago. We present also completely independent results for precipitation and selected atmospheric indices.

Analysis skill is assessed with the following measures: First we calculate the Pearson correlation coefficient to evaluate the covariability of data sets. Second, we use the Reduction of Error (RE) statistic^74^, also known as the mean squared error skill score^75^. RE is calculated with the following equation:

(9)RE=1−∑(xia−xiref)2∑(xib−xiref)2

where *x*^*a*^ is the analysis EKF400, *x*^*b*^ is a ‘no knowledge prediction’ and *x*^*ref*^ is the reference, in our case the CRU TS 3.10 instrumental data. As no knowledge prediction we choose CCC400 in which case values above 0 indicate that EKF400 is closer to CRU TS3.10 than CCC400 and hence the assimilation step has added information. Note that RE uses squared absolute deviations, i.e., RE can be negative even if correlations are strongly positive.

**Figure 4:**
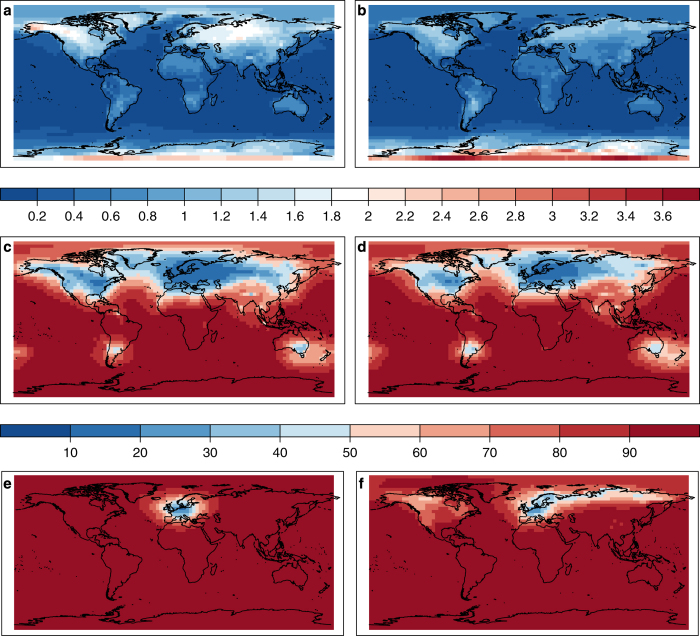
The data assimilation reduces the temperature spread of the 30 CCC400 ensemble members. The original CCC400 spread is shown for October to March (**a**) and April to September (**b**). The remaining percentage of ensemble spread after the data assimilation can be seen during the verification period 1902–2001 in (**c**, **d**) and for 1651–1750 when mostly tree-ring measurements are assimilated in (**e**, **f**). The October to March season is shown in the left column (**a**, **c**, **e**) and the April to September season in the right column (**b**, **d**, **f**).

The ensemble-based error estimate is a major advantage of our assimilation in comparison with previous statistical reconstruction approaches. We highlight how the standard deviation, often called ‘spread’, of the ensemble simulations is reduced by the assimilation (Fig. 4). This alone, however, is not enough to judge the reliability of the system. Reliability can be evaluated with the spread-error ratio (SPR2ERR):

(10)SPR2ERR=nens+1nens⋅s¯ens2MSE

Here, the temporal mean of the ensemble variance $\left({\bar{s}}_{\mathrm{ens}}^{2}\right)$ is divided by the mean squared error of the observations from the ensemble mean (MSE). The intra ensemble sample variance is inflated to take the finite ensemble size (*n*_ens_) into account^75^. Values of SPR2ERR close to 1 indicate an appropriate ensemble spread and consequently the observations are consistent with the analysis and analysis error estimate. Values of SPR2ERR smaller than 1 indicate overconfidence with insufficient ensemble spread. In our assimilation, overconfidence can be caused by a variety of factors including insufficient localization, underestimation of observation errors, correlation of observation errors, or insufficient spread in CCC400. Values of SPR2ERR larger than 1 on the other hand indicate overdispersion.

This SPR2ERR definition assumes error-free observations. Errors in observations can be treated as additive noise^76,77^. Our error adjusted MSE_a_ is given by:

(11)MSEa=MSE−SDobs2

where $SD_{\mathrm{obs}}^{2}$ is the variance of the CRUTEM4 ensemble estimate of gridded instrumental temperature observations^16^.

To evaluate the reanalysis skill before the 20th century and its variation over time, we conduct a leave-one-out (LOO) validation for the period from 1760 to 1880. We systematically discard instrumental temperature observations of a single grid box and compare the resulting analysis with the independent, discarded measurements. This gives us two important pieces of information. First, at each grid cell with measurements, we can calculate the correlation and MSE with independent data. Second, with the ensemble spread of the LOO analysis and the MSE we can calculate the SPR2ERR further back in time. In contrast to the 20th century, when information was spatially nearly complete, in the earlier period we only have information at a few locations with observations. Thus, the temporal evolution of SPR2ERR change only represents this area with observations.

### Reanalysis skill

First, we assess the skill with a focus on the 100yr period 1902–2001 CE, when the overlap with instrumental observations allows for comparisons. We evaluate EKF400 with CRU TS3.10 as a reference.

Within the CCC400 ensemble we find the largest temperature spread in continental regions and high latitudes during the winter season of each hemisphere (Fig. 4, top). Data assimilation reduces the ensemble spread with respect to measurement uncertainty where the covariance matrix indicates spatial relationships within the localization distance. In regions with considerable spread and with a dense network such as Europe, the spread can be reduced to 10–20% of the original CCC400 spread whereas it remains unchanged at nearly 100% distant from any observation, e.g., in central Africa (Fig. 4, middle). Looking back into the period 1651–1750 of EKF400, when only European documentary information is assimilated in October to March, the spread reduction is limited to Europe. During the April to September season, when tree-ring data is available in most extra-tropical land regions of the northern hemisphere spread can additionally be reduced in northern Russia and northern North America (Fig. 4, bottom).

**Figure 5:**
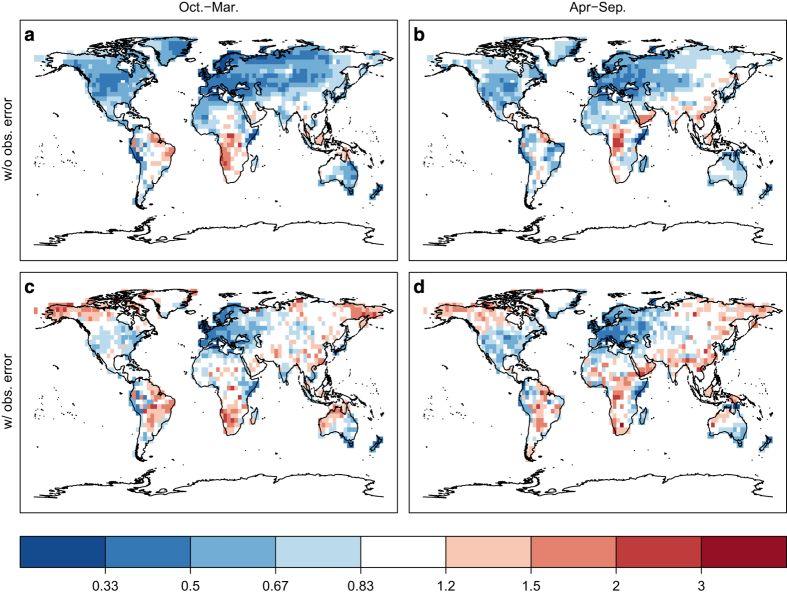
The spread to error ratio of EKF400 during the 20th century indicates where and when the analysis is overdisperse or overconfident. The maps at the top (**a**, **b**) result from the assumption of perfect observation whereas errors in observations are accounted for in **c**, **d**.

The SPR2ERR for the 20th century suggests that overfitting (too much reduction of model spread without comparable reduction of error) occurs in the 20th century in large parts of North America and Eurasia (Fig. 5, top). If uncertainties in the observations are considered, we only find clear overfitting in Europe and slight overfitting in the United States of America (Fig. 5, bottom). This could have two potential reasons. First, the spread of CCC400 is already too small because of the relatively small ensemble size of 30 members. This can be ruled out by the spread-error analysis for CCC400 (not shown) that indicates too little spread at very few coastal grid boxes in the tropics, which are most likely a result of the SST forcings. Second, the observation error is too small or correlated. Unfortunately, there is no quality information for most of the records and we have only been able to assign a best guess error from the literature to all series. Additional analysis suggests that representativity errors may be larger than prescribed as our grid boxes span a larger area than in the study of MeteoSwiss^24^. The challenge of assigning adequate observation errors requires further investigation in the future. In contrast to Europe and North America, spread-error ratios for central South America and central Africa indicate that the model spread could not be reduced sufficiently.

**Figure 6:**
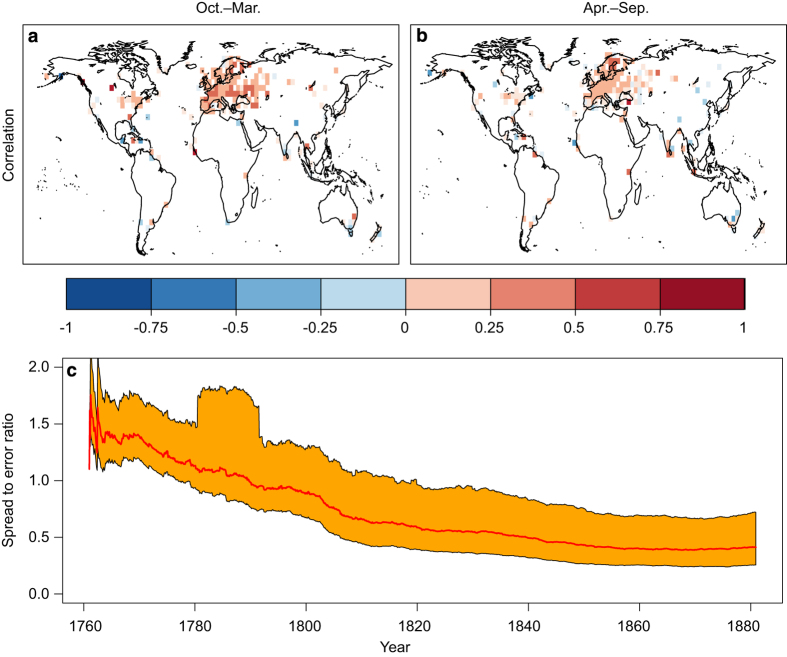
The EKF400 skill in the period 1761–1880 is assessed with a leave-one-out validation. The maps (**a**, **b**) show the Pearson correlation coefficients between each excluded and thus independent observation and the analysis with all remaining observations assimilated. The October to March season is shown on the left (**a**), April to September on the right (**b**). The time series in (**c**) highlights the evolution of the median spread to error ratio (red) and the 0.25 to 0.75 quartile range (orange) of the leave-one-out experiment as 11-year moving averages.

SPR2ERR from the LOO experiments confirm the results from the 20th century. When hardly any instrumental temperature data is assimilated such as from 1760 to 1770, median SPR2ERR values of 1.5 indicate overdispersion (Fig. 6e). From 1780 onward the SPR2ERR decreases with median values reaching optimal ratios of one around 1800 and asymptotically approaching values of 0.5 indicating overconfidence at the end of the 19th century. The main reason for overconfidence is a reduction in ensemble spread over central and eastern Europe without a significant reduction in MSE. The fact that assimilating few measurements leads to a desired SPR2ERR ratio of one but a denser data network to ratios below one suggests that measurement errors are currently underestimated. Hence, future methodological improvements will focus on finding more data in sparse regions and further back in the past as well as improvements in assigning measurement errors, localisation distances and observation error correlation.

**Figure 7:**
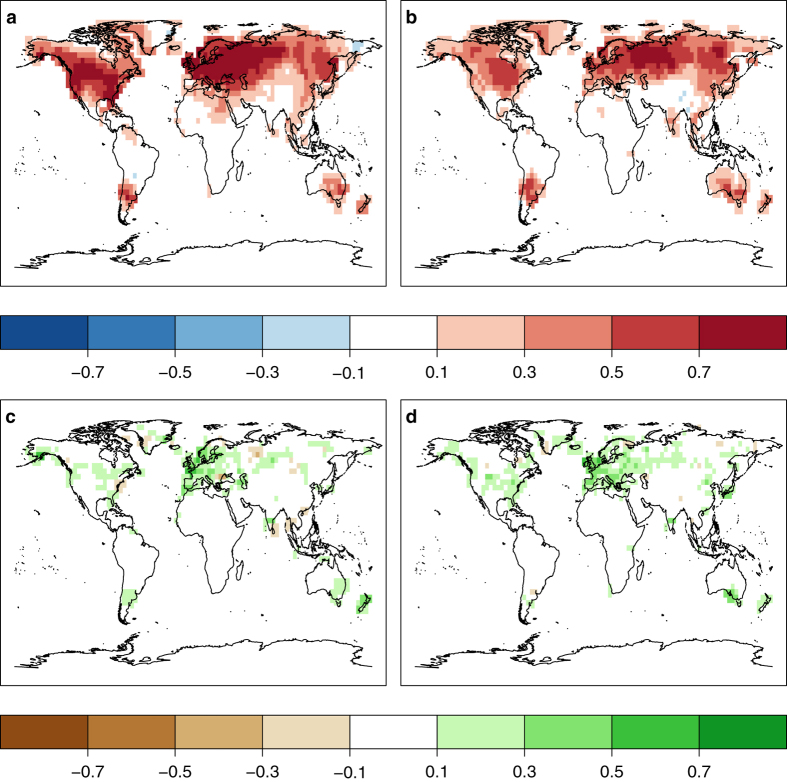
The increase in correlation highlights the improvements due to the assimilation procedure. The maps show differences of correlations between gridded instrumental CRU TS 3.10 data and CCC400 and EKF400. Temperature is presented on top (**a**, **b**), precipitation at the bottom (**c**, **d**). The October to March season is shown on the left (**a**, **c**), April to September on the right (**b**, **d**).

Ensemble mean simulated temperature and observed temperatures are positively correlated already before data assimilation has been applied, in particular in coastal regions. The main reason is the model forcing and here specifically the prescribed sea surface temperatures. Data assimilation, however, greatly enhances positive correlation between EKF400 and instrumental CRU TS3.10 data, as seen in the correlation differences before and after the assimilation (Fig. 7a,b). Only in limited regions of central South America, central Africa and the Middle East, which are far away from any observation, we find no improvement in correlation. Although we do not assimilate any precipitation data, EKF400 has much higher correlations with instrumental precipitations measurements than CCC400 (Fig. 7c,d) due to covariances between temperature/SLP and precipitation. This increase in correlation is strongest in Europe and the United States, where most data gets assimilated, but improvements can also be seen in southern South America Southwest Australia and New Zealand. LOO experiments confirm the increase in correlation between independent validation data and the analysis in almost all location for the period 1761–1880. Highest correlation coefficients are reached in Europe and the eastern United States of America where the observation network is densest and nearby observations can substitute the observation that has been left out (Fig. 6a,b).

**Figure 8:**
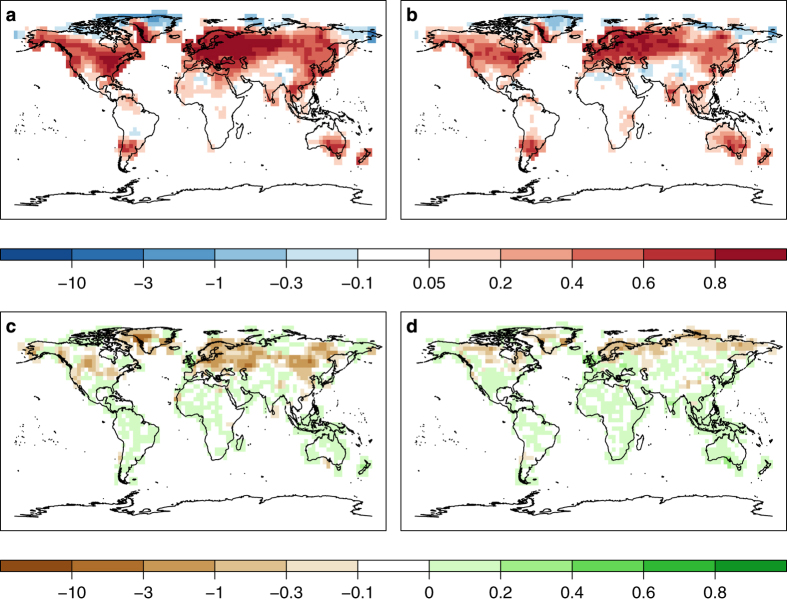
Positive values of the reduction of error (RE) statistic indicate that the reanalysis is closer to the validation data than the CCC400 ensemble mean. RE for temperature and the seasons October to March (**a**) and April to September (**b**) and precipitation (**c**, **d**).

If the RE is used to evaluate improvements of EKF400 ensemble mean over CCC400 ensemble mean, we similarly find positive skill in temperature for most of the northern hemisphere apart from central/northern Greenland and eastern Russia (Fig. 8a,b). In these regions, however, negative values may actually mean an improvement, because the instrumental data set that is used for validation has too little variability at grid cells far away from any station where values were either interpolated or long term averages have been filled in. This is the case, for example, over central Greenland, where CRU TS3.10 has basically no variability. In theory, spurious correlation in the covariance matrix, which is based on 30 ensemble members only, could lead to such errors in the analysis but in general the localization prevents such corrections. In regions far away from any assimilated data, e.g., in Africa, RE values indicate zero skill, i.e., EKF400 does not differ from CCC400. RE values in the LOO experiments for the years 1761–1880 are globally around zero (not shown). The reason is not that the LOO analysis ensemble mean would be equal to the CCC400 ensemble mean, LOO has clearly enhanced variance. Although the amount of variance itself is closer to the variance of the observations, it causes the low RE values because both overestimation and underestimation of the observed value get punished in the RE statistic. That is why the low variability CCC400 ensemble mean can achieve the same RE values than the LOO ensemble mean although LOO variability and correlation indicate that the LOO analysis is superior to CCC400.

The RE values for precipitation are negative in some regions of Europe, Asia and North America, indicating that the assimilation of temperature and SLP is not sufficient to lead to an improvement in absolute precipitation amounts. Additional experiments that assimilated precipitation measurements did not lead to better skill in EKF400 precipitation, either. Precipitation is locally very variable and not spatially correlated over larger regions. Due to regional covariability between temperature/SLP and precipitation, EKF400 mostly has the correct sign of the precipitation anomaly (Fig. 7) but not the correct amount (Fig. 8).

**Figure 9:**
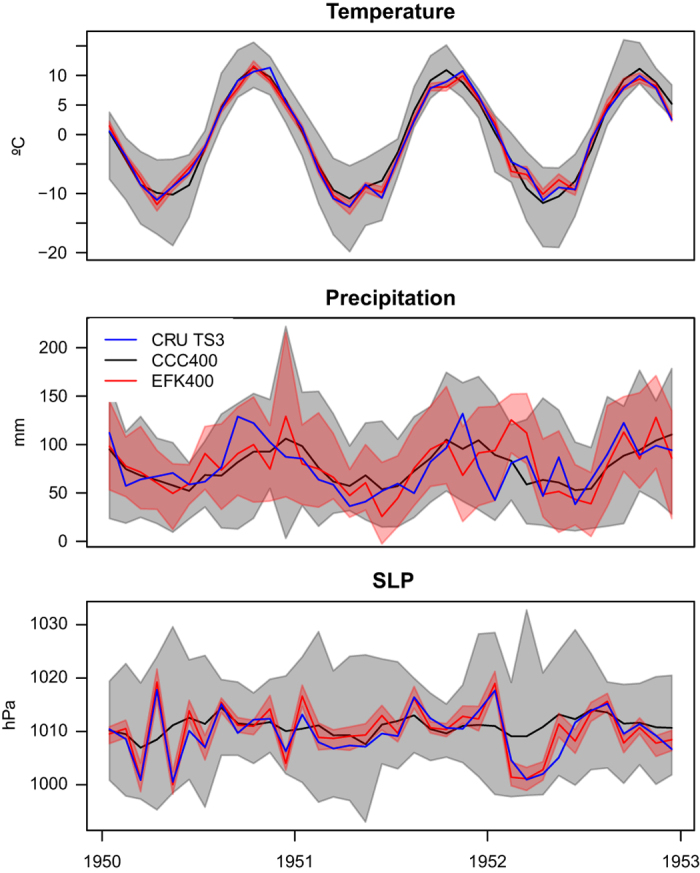
These examples highlight how the assimilation affects the final reanalysis. The time series are monthly averages for 3 years in Norway (62° N, 11° E) for temperature (top), precipitation (middle) and sea level pressure (bottom), all include instrumental data (blue), CCC400 ensemble mean/spread (black/grey) and EKF400 ensemble mean/spread (red/light red).

How the assimilation works and what differences in correlation and RE mean, can be best understood in an example. The monthly average temperature at a grid box in Norway (62° N, 11° E) in the CCC400 ensemble mean shows too little variability, has a correct annual cycle, but does not correlate with instrumental data, if the annual cycle is removed, because of internal variability in the model (Fig. 9). The assimilation of temperature and pressure measurements leads to a nearly perfect match between instrumental temperature and EKF400. For precipitation, the CCC400 ensemble also has too little variability. Here the assimilation also causes an increase in variability, mostly pointed in the correct direction. However, this increase is often too large, which causes the absolute values of EKF400 being further away from instrumental data than CCC400. For SLP the assimilation works nearly as well as for temperature. We also see the overconfidence suggested by the spread-error ratio in Fig. 5, because some of the temperature and SLP values lie outside the narrow band of EKF400 spread.

### Northern hemisphere temperature variability

One important aspect of paleoclimatology has long been the reconstruction of hemispheric to global temperature variability to place the current global warming into a longer-term perspective. Although a lot of progress has been made both in methodology and the amount of paleodata records for single locations, substantial uncertainties remain until today. These are often related to the selected set of records, the applied statistical method, seasonality and their regional representativity^78^.

**Figure 10:**
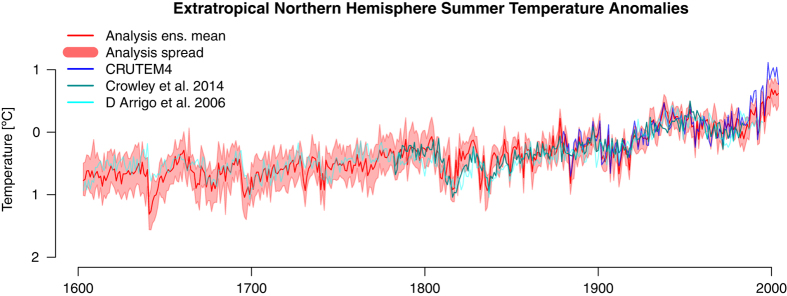
Northern hemisphere temperature evolution over the past centuries is a key figure in many paleoclimatic reconstructions. Here we present average extratropical northern hemisphere summer land temperature anomalies (wrt 1901–1980) 2 m above the surface EKF400 ensemble mean and members (dark and light red), CRUTEM4 gridded instrumental data (blue) and selected reconstructions (light and dark cyan).

Large-scale temperature averages vary from annual to centennial and longer time scales due to internal weather processes and external forcings. In Fig. 10 extratropical northern hemisphere (ENH, 20–90° N) summer (April to September) land temperature variability has been plotted from 1600 to 2005 CE. because that is what northern hemisphere temperature limited tree-rings record, in which play the dominant role in most previous reconstructions. Additionally, this is the region and season, in which EKF400 is expected to have most skill, especially in the early part when mostly tree-ring data are assimilated. The light red band in Fig. 10 highlights the spread of the EKF400 ensemble. It has a spread of roughly 0.7 K before 1800 when mainly tree-ring data is assimilated and a spread decreasing to ~0.3 K after 1800 with the increase in the number of instrumental measurements. The co-evolution of the EKF400 ensemble mean, recent proxy-based reconstructions and instrumental data sets indicate clear improvements due to the assimilation (Table 3). To remove the influence of long term trends or multi-decadal variability on the correlation coefficient, we calculate correlations not only based on the original series but additionally on a 11-year high pass filtered version, in which low frequency variability has been removed. The results highlight that CCC400 is highly correlated with the reference series because of common multi-decadal changes prescribed by the forcings. On sub-decadal time scales EKF400 correlates better with the instrumental data and paleodata reconstructions. The skill in EKF400 is also visible in the clearly positive RE values with a maximum of 0.79 if compared to CRUTEM4 ref.(16). Similar to the global mean, we also find high agreement between continental scale temperature reconstructions of the PAGES 2k Consortium^79,80^. Note, the underestimation of ENH average temperature in the last 35 years is an artefact of using 70 year average anomalies with respect to the current year. For the past 35 years, we had to shorten this period and keep the same final year in the calculation of the anomalies.

The ensemble standard deviation of northern hemisphere extra-tropical land temperature in CCC400 is 0.35/0.19 (Oct-Mar/Apr-Sep) due to internal variability in the model. EKF400 has a standard deviation of 0.32/0.18 between 1600–1800 CE. Values decrease rapidly in the first 20 years of the 19th century down to 0.13/0.09. In comparison, the CRUTEM4 ensemble suggests observation and interpolation errors in the instrumental data with values of 0.11/0.09 is very close to the level that EKF400 reaches at the early 19th century.

### Case study: 1816, the ‘year without a summer’ in Europe after the eruption of Mt. Tambora

To look a bit closer into the performance of EKF400 in earlier times and at the possibilities it offers for understanding the climate dynamics, we present a case study for the summer 1816 in Europe, which followed the Tambora eruption in April a year earlier and which led to the last subsistence crisis in central Europe^81^. For the case of Geneva, Switzerland, only about half of the cooling could be related to the volcanic aerosol forcing and the other half was related to more frequent westerlies, which brought rain from the Atlantic to central Europe^82^.

**Figure 11:**
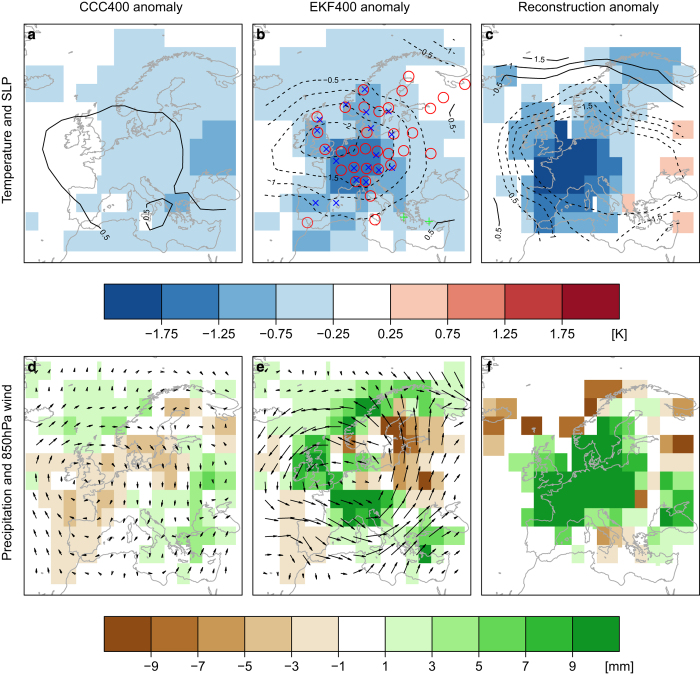
A great advantage of paleo-reanalysis over statistical reconstructions is the possibility the interpret the multivariate state of the atmosphere. Temperature and SLP anomalies for April to September 1816 in CCC400 (**a**), EKF400 (**b**) are compared to the reconstructions by Luterbacher *et al.* 2004 and Küttel *et al.*^46^ (**c**), precipitation and 850 hPa wind anomalies in CCC400 (**d**), EKF400 (**e**) to the reconstruction by Pauling *et al.*^64^ (**f**).

In the CCC400 simulations, which are forced with the reconstructed aerosols, we find a cooling of ca. 0.5 K over Europe and neither strong anomaly in SLP nor precipitation (Fig. 11a,d). The assimilation leads to a much stronger temperature anomaly of around −1.5 K in central Europe and a SLP anomaly of −2 hPa (Fig. 11b). These values in the EKF400 ensemble mean are very close to the reconstructions of temperature^63^ and SLP^46^ where anomalies of −2 K and −2 hPa have been found (Fig. 11c). Interestingly, EKF400 suggests no warming, but normal temperatures in Eastern Europe, while reconstructions indicate warming.

The latter two data sets are partly based on the same input data. This is not the case for precipitation. EKF400 shows a strong positive precipitation anomaly over central Europe (Fig. 11e), similar to reconstructed precipitation^64^ (Fig. 11f). Beyond this, EKF400 also highlights the enhanced westerlies in the wind anomaly field, which have been described based on early observations^82^ (Fig. 11b).

### Atmospheric indices

**Figure 12:**
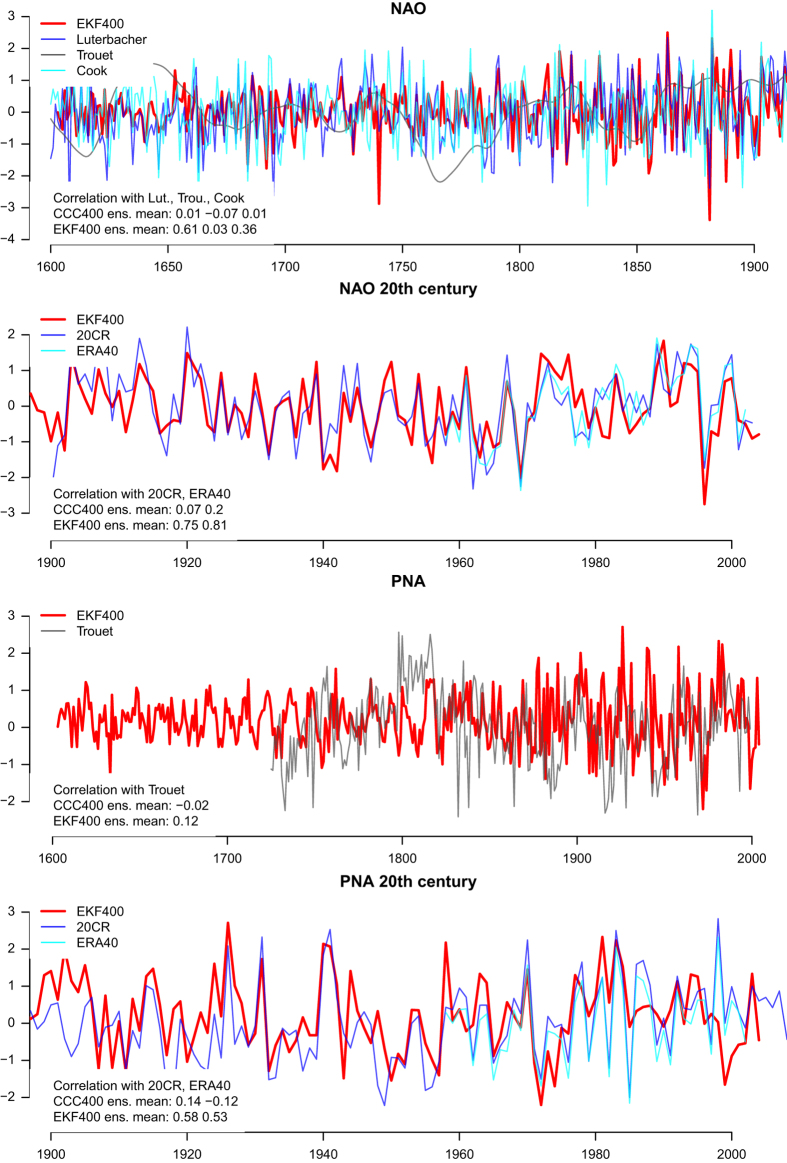
The availability the full atmospheric state also allows for the calculation of circulation indices. The December to March NAO/PNA index (top/bottom) of EKF400 correlates much better with the reference series during the 20th century (top) than CCC400. Values in the lower left corner indicate correlation coefficient of EKF400/CCC400 with the reference data sets for the overlapping period.

EKF400 allows for deriving large-scale averages or indices from its spatial fields. With the current setup and paleodata network there is skill in the North Atlantic Oscillation index (NAO) and the Pacific North American pattern (PNA) (Fig. 12), both calculated as annual averages over the months December to March. Skill in the NAO increases from near zero correlation between CCC400 and 20CR^2^ and ERA40 ref.(73) to correlations of around 0.8 with EKF400. This may come to less of a surprise because SLP data from Iceland and Portugal gets assimilated. However, during the period from 1600–1900 correlations with NAO reconstructions^68,69^ increase from zero in CCC400 to 0.36/0.61 in EKF400, respectively, although few SLP observations are assimilated in the 19th century and none before. Only the low frequency reconstruction by Trouet *et al.*^70^ appears to disagree with all the other reconstructions including EKF400.

PNA is calculated from geopotential height fields at 500hPa and skill in this region solely stems from the assimilation of a few temperature observations and the many tree-ring measurements. Here, correlations with 20CR^2^ and ERA40 ref.(73) in the 20th century increase from around 0 for CCC400 to ca. 0.55 for EKF400. However, we neither find any agreement between EKF400 and a previous reconstruction^71^ in the 20th century nor before. In pseudoproxy experiments^6^ skill has been found in reconstructing the strength of the polar vortex, the Hadley cell, the position of the subtropical jet and the dynamic monsoon index. Out of these, we currently only see skill in the polar vortex strength at 100 hPa. For more skill at lower latitudes, our current paleodata network and covariance matrix together with the applied localization do not provide sufficient constraints.

## Usage Notes

Combing direct and indirect climate observations, climate forcing time series and a climate model, this data set joins all our information including their uncertainties into the currently best estimate of monthly variability of the three-dimensional atmosphere during the past 400 years. A few thousand kilometres around assimilated data, EKF400 contains more realistic information than the unconstrained model ensemble. Hence, this new paleo-reanalysis will mainly allow for new insights around northern hemisphere land areas whereas oceanic regions closely follow the SST forcings.

The EKF400 reanalysis described in this article is available at the World Data Center for Climate at Deutsches Klimarechenzentrum (DKRZ) in Hamburg, Germany (Data Citation 1). A login is provided to everybody after sending an email to ‘data@dkrz.de’.

Note, the data south of 60°S should not be used (see ‘model simulation’ section). In general, EKF400 has little skill in the southern hemisphere that goes beyond the original model simulations because only few observations have been assimilated.

Variability on time scales longer than 70 years stems from the model and its forcings, including SST forcings. This data set should therefore not be used to analyse multi-decadal variability of oceanic indices.

The assimilation is conducted based on anomalies. If the mean is added to receive absolute values, this may rarely result in total precipitation values below zero. Please be aware of this artefact.

Future improvements should include an extension of the paleodata network and better error assessment of the assimilation data. Largest gains in analysis skill can be expected from an improved description of the background covariance matrix, which is currently only based on the spread of the model ensemble at the assimilation time. An improved background covariance matrix would reduce the need for localization and hence allow accounting for more distant teleconnections.

## Additional Information

**How to cite this article**: Franke, J. *et al.* A monthly global paleo-reanalysis of the atmosphere from 1600 to 2005 for studying past climatic variations. *Sci. Data* 4:170076 doi: 10.1038/sdata.2017.76 (2017).

**Publisher**’**s note**: Springer Nature remains neutral with regard to jurisdictional claims in published maps and institutional affiliations.

## Supplementary Material



## Figures and Tables

**Table 1:** Variables in the state vector and their decorrelation distances.

**Variable**	**Decorrelation distance (km)**
Temperature (2 m)	1,500
Temperature (500 hPa)	1,500
Sea level pressure	2,700
Precipitation	450
Geopotential height (500 hPa)	2,700
Geopotential height (100 hPa)	3,750
Wind (850 hPa, longitudinal component)	1,800
Wind (850 hPa, latitudinal component)	1,800
Wind (200 hPa, longitudinal component)	1,500
Wind (200 hPa, latitudinal component)	1,500
Vertical motion (Omega, 500 hPa)	750

**Table 2:** Data sets used and generated in this study.

**Name**	**Type**	**Period**	**Temporal resolution**	**Area**	**Spatial resolution**	**Variables**	**Reference**
CCC400	ECHAM5.4 all forcing simulation	1600–2005	6-h	Global	2°×2°	All ECHAM output variables	Bhend *et al.*^6^
EKF400	Assimilation (30 ensemble members)	1600–2005	Monthly	Global	4°×4°	Temperature, precipitation, sea level pressure, wind, geopotential height, vertical motion	This study

**Table 3:** Pearson correlation coefficients (r) between CCC400/EKF400 and instrumental data/selected reconstructions, all for the extra-tropical northern hemisphere land temperature mean.

	**r CRU** **before/after 11-year high pass filtering**	**r Crowley before/after 11-year high pass filtering**	**r D’Arrigo before/after 11-year high pass filtering**	**RE CRU**	**RE Crowley**	**RE D’Arrigo**
CCC400	0.76/0.27	0.85/0.19	0.81/0.12	0.79	0.18	0.37
EKF400	0.85/0.81	0.51/0.37	0.48/0.26			
The first value is based on the original time series including all frequencies and after applying an 11-year high-pass filter to focus on improvements in inter-annual variability that is less influenced by external forcings. RE values indicate how much closer EKF400 is to the reference than CCC400. Always the entire overlapping period of the series is considered (CRU: 1850–2004; Crowley: 1782, 1984; D’Arrigo: 1608–1990).						
